# Csk-Induced Phosphorylation of Src at Tyrosine 530 is Essential for H_2_O_2_-Mediated Suppression of ERK1/2 in Human Umbilical Vein Endothelial Cells

**DOI:** 10.1038/srep12725

**Published:** 2015-08-03

**Authors:** Bo Kyung Jeon, Kihwan Kwon, Jihee Lee Kang, Youn-Hee Choi

**Affiliations:** 1 Department of Physiology, School of Medicine, Ewha Womans University, Seoul, Korea; 2 Tissue Injury Defense Research Center, School of Medicine, Ewha Womans University, Seoul, Korea; 3 Department of Internal Medicine, Division of Cardiology, School of Medicine, Ewha Womans University, Seoul, Korea

## Abstract

Mitogen-activated protein kinases (MAPKs) are key signal transducers involved in various cellular events such as growth, proliferation, and differentiation. Previous studies have reported that H_2_O_2_ leads to phosphorylation of extracellular signal-regulated kinase (ERK), one of the MAPKs in endothelial cells. The current study shows that H_2_O_2_ suppressed ERK1/2 activation and phosphorylation at specific concentrations and times in human umbilical vein endothelial cells but not in immortalized mouse aortic endothelial cells or human astrocytoma cell line CRT-MG. Phosphorylation of other MAPK family members (i.e., p38 and JNK) was not suppressed by H_2_O_2_. The decrease in ERK1/2 phosphorylation induced by H_2_O_2_ was inversely correlated with the level of phosphorylation of Src tyrosine 530. Using siRNA, it was found that H_2_O_2_-induced suppression of ERK1/2 was dependent on Csk. Physiological laminar flow abrogated, but oscillatory flow did not affect, the H_2_O_2_-induced suppression of ERK1/2 phosphorylation. In conclusion, H_2_O_2_-induced Csk translocation to the plasma membrane leads to phosphorylation of Src at the tyrosine 530 residue resulting in a reduction of ERK1/2 phosphorylation. Physiological laminar flow abrogates this effect of H_2_O_2_ by inducing phosphorylation of Src tyrosine 419. These findings broaden our understanding of signal transduction mechanisms in the endothelial cells against oxidative stress.

Reactive oxygen species (ROS) such as superoxide, hydrogen peroxide (H_2_O_2_), and the hydroxyl radical are generated by all aerobic cells. Low-dose ROS act as signaling molecules and regulate both gene expression and signal transduction^1^,^2^,^3^,^4^. However, when excess ROS overwhelm endogenous antioxidant systems, oxidative stress occurs, leading to harmful effects such as aging, senescence, and apoptosis^5^,^6^. In endothelial cells, excessive ROS mediate vasodilation, endothelial barrier dysfunction, actin reorganization, and leukocyte extravasation^7^,^8^,^9^,^10^,^11^. ROS are abundant, especially in the endothelium at sites of inflammation and infection, and are generated by various sources including vasoactive peptides, cyclic stretch, hypoxia-reoxygenation, and infiltration of activated leukocytes^12^. The pathogenesis of diverse vascular diseases, including atherosclerosis, diabetes, and ischemia-reperfusion injury, is associated with the overproduction of ROS^8^.

Endothelial cells that line the inner surface of blood vessels are continually exposed to various stresses, including oxidative stress and mechanical force induced by blood flow and pressure. Laminar shear stress, one of the primary forces that endothelial cells experience, is particularly important because it is critical to endothelial cell survival and proliferation, and the regulation of various genes such as heme oxygenase, connexin 37, and growth arrest and DNA damage-inducible protein 153^13^,^14^. However, when unidirectional shear flow is disrupted, the gene expression profile is changed and pro-atherogenic signaling is activated^15^,^16^. When endothelial cells are exposed to oxidative stress, signaling molecules such as extracellular signal-regulated kinases (ERKs), protein kinase C, and tyrosine kinase are activated^8^. Increasing ERK1/2 activation in endothelial cells by ROS influences several endothelial activities such as pro-survival effects against apoptosis, increased vascular permeability, and MMP-9-mediated angiogenesis^7^,^17^,^18^.

The mitogen-activated protein kinases (MAPKs) are a family of serine-threonine kinases that act as key signaling molecules regulating numerous cellular functions^19^. Responding to both extracellular and intracellular stimuli such as growth factors, cytokines, and oxidative stress, MAPKs phosphorylate specific serine and threonine residues on target proteins and control cellular activities including gene expression, metabolism, proliferation, and apoptosis^20^. ERKs, c-Jun NH2-terminal kinases (JNKs), and p38 enzymes are major subfamilies of MAPKs. However, these subfamilies have somewhat different effects on various eukaryotic cells. In general, ERKs regulate cell survival and proliferation in differentiated cells, while JNK controls the apoptotic response to cellular stress and p38 plays a critical role in normal immune and inflammatory responses^21^,^22^,^23^. Dysregulation of ERK1 and ERK2 (ERK1/2) signaling pathways is related to various human diseases including neurodegenerative diseases, cancers, and inflammation^24^. Previous studies have reported that extracellular H_2_O_2_ leads to phosphorylation of ERK1/2 and activation of the ERK signaling pathway in various cell types, including fibroblast, smooth muscle, and endothelial cells^8^,^25^,^26^. ERK1/2 regulates cell functions by phosphorylating many cytosolic proteins such as p90 ribosomal s6 kinase, MAPK-interacting serine/threonine kinase, and the transcription factor Elk^27^. MAPK kinase (MAPKK), MEK1/2, and MAPKK kinase (MAPKKK; A-Raf and B-Raf) are upstream activators of ERK1/2. Their activation is triggered by G protein-coupled receptors, receptor tyrosine kinases, and non-receptor tyrosine kinases such as Src^28^,^29^.

Src is an oncogene that is overexpressed in many types of tumors^30^. In endothelial cells, Src activates several molecules that are involved in endothelial permeability and induce vascular leakage^31^. Src is regulated at two major tyrosine phosphorylation sites (419 and 530). When phosphorylation occurs at tyrosine 419, Src undergoes a conformational change so that substrates are able to access the Src kinase domain. However, when Src is phosphorylated at tyrosine 530, kinase activity is blocked^32^. Csk is a negative regulator of Src that phosphorylates Src at tyrosine 530 and maintains the inactive state^33^,^34^.

The present study was designed to determine the molecular mechanisms by which H_2_O_2_ suppresses p-ERK1/2 phosphorylation, focusing on the Csk-Src-ERK signaling pathway. The results demonstrate that phosphorylation of ERK1/2 is suppressed over a specific concentration range of H_2_O_2_ in human umbilical vein endothelial cells (HUVECs). The study also found that physiological laminar shear stress could modulate this suppression.

## Results

### H_2_O_2_ reduces the endogenous level of ERK1/2 phosphorylation

**Figure 1:**
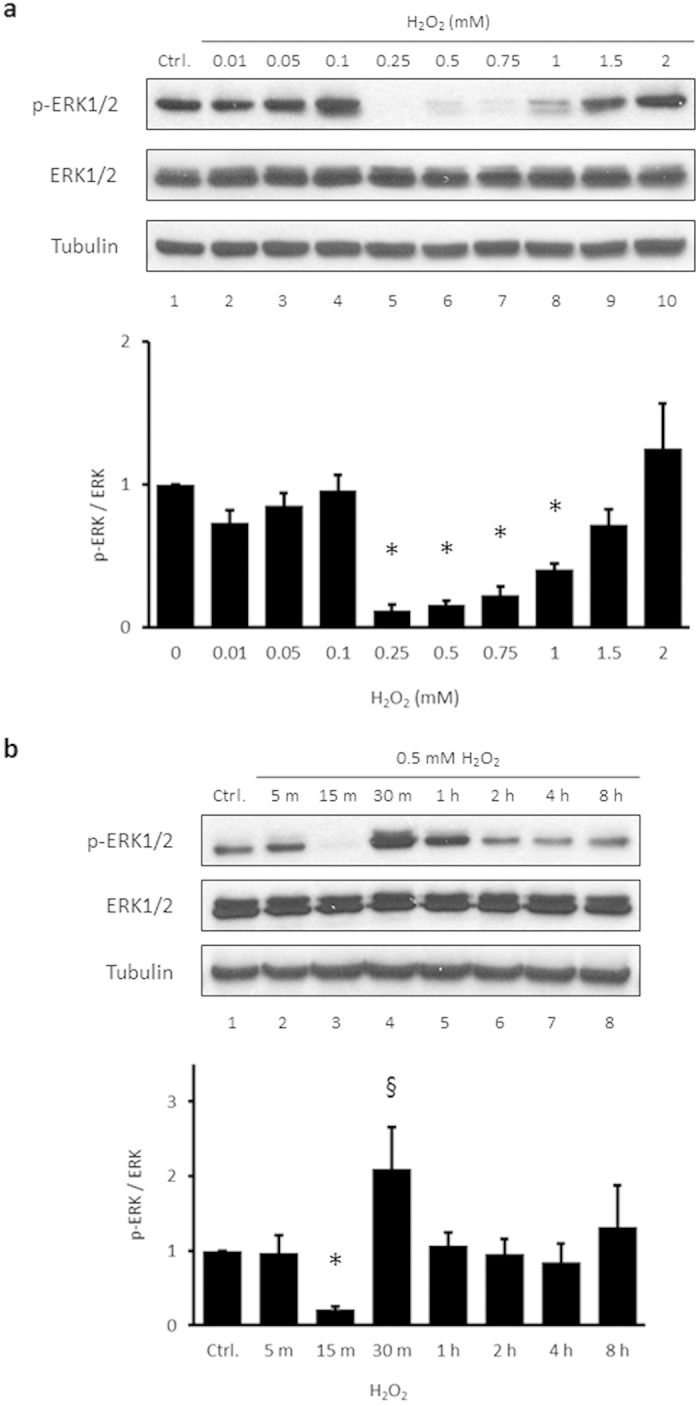
Phosphorylation of ERK1/2 in HUVECs is suppressed by treatment with H_2_O_2_. (**a**) HUVECs were treated with 0–2 mM H_2_O_2_ for 15 min. Cells were washed once with PBS. Whole cell lysates were then extracted and analyzed by immunoblotting. Anti-p-ERK1/2, anti-ERK1/2, and anti-tubulin antibodies were used. Tubulin was used as the loading control. Bottom panel: p-ERK1/2 levels were quantified densitometrically and normalized to the level of ERK1/2. The control level of p-ERK1/2 expression was assigned a value of 1. Data are expressed as mean values ± SEM of three independent samples. *p < 0.05 vs. untreated control. (**b**) HUVECs were treated with 0.5 mM H_2_O_2_ for the indicated times (5 min to 8 h). Whole cell lysates were extracted and analyzed by immunoblotting. Data are expressed as mean values ± SEM of three independent samples. *p < 0.05 vs. untreated control; ^§^*p* < 0.05 between 15 min- and 30 min-treated cells. Ctrl, untreated control; p, phospho.

HUVECs were treated with various concentrations of H_2_O_2_ for 15 min and examined for changes in ERK1/2 phosphorylation by immunoblotting. In the presence of 0.25–1 mM H_2_O_2_, ERK1/2 phosphorylation was significantly reduced compared to the untreated control group, with no change in the total amount of ERK1/2 (Fig. 1a). However, when 2 mM concentrations of H_2_O_2_ were tested, ERK1/2 phosphorylation was slightly increased. Treating HUVECs with a mid-range concentration of H_2_O_2_ (0.5 mM) for varied time periods showed that p-ERK1/2 was slightly increased at 5 min but significantly reduced at 15 min (Fig. 1b). Interestingly, ERK1/2 phosphorylation was increased at 30 min after H_2_O_2_, compared to the 15 min-treated and control groups. After 1 h, ERK1/2 phosphorylation was decreased to levels comparable to the control.

### H_2_O_2_ reduces ERK1/2 phosphorylation but has little effect on the phosphorylation of p38 and JNK

**Figure 2:**
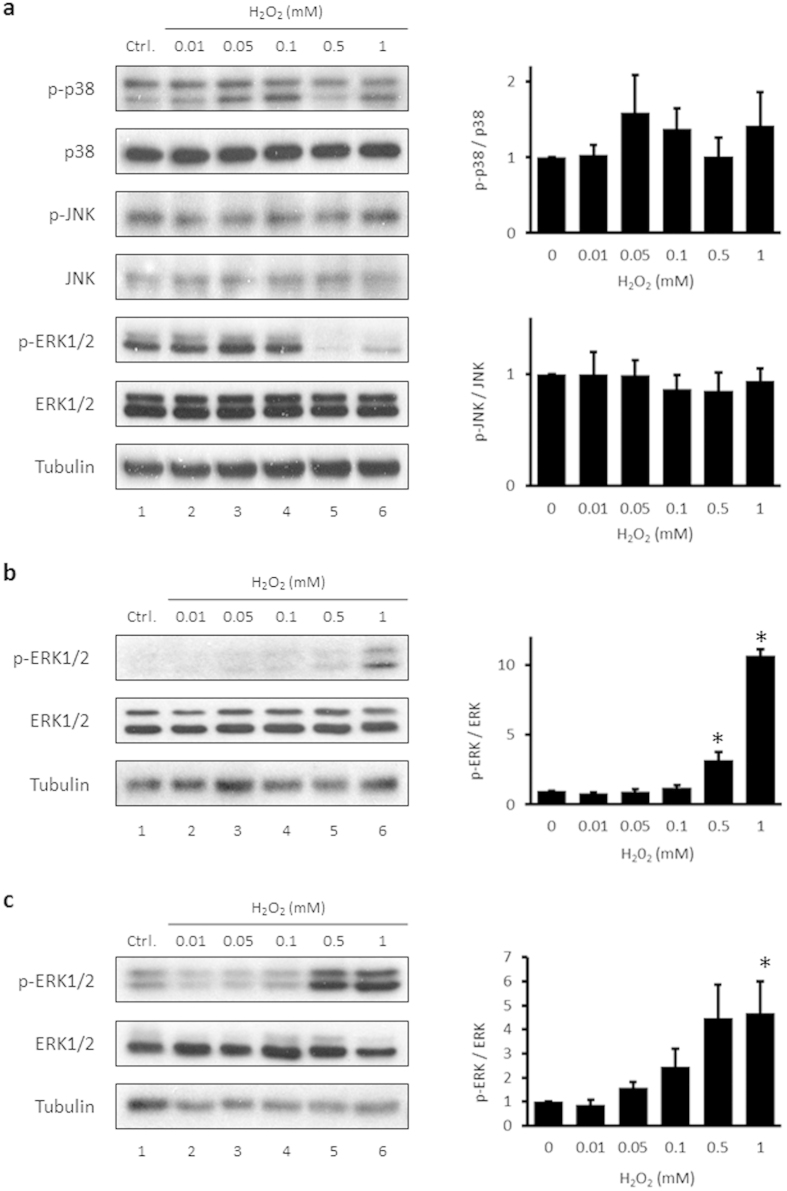
H_2_O_2_-mediated suppression of ERK1/2 is specific to HUVECs. (**a**) HUVECs were treated with 0–1 mM H_2_O_2_ for 15 min. Whole cell lysates were extracted and analyzed by immunoblotting. Antibodies against p-p38, p38, p-JNK, JNK, p-ERK1/2, ERK1/2, and tubulin were used. Tubulin was used as the loading control. Right panel: p-p38 and p-JNK levels were measured densitometrically and normalized to the levels of p38 and JNK, respectively. The control level was assigned a value of 1. Data are expressed as mean values ± SEM of three independent samples. Varied concentrations of H_2_O_2_ were used to treat (**b**) iMAEC and (**c**) CRT-MG cells for 15 min. Whole cell lysates were extracted and analyzed by immunoblotting. Data are expressed as mean values ± SEM of three independent samples. *p < 0.05 vs. untreated control. p, phospho.

ERK1/2, p38, and JNK influence each other in response to various stresses^35^. To test whether these MAPK subfamilies are affected by H_2_O_2_, HUVECs were treated with varied concentrations of H_2_O_2_ for 15 min and the phosphorylation of p38 and JNK was examined by immunoblotting. Treating HUVECs with 0.5 or 1 mM H_2_O_2_ decreased p-ERK1/2 but had no effect on p-p38 (Fig. 2a). Interestingly, phosphorylation of p38 was slightly increased with 0.05 mM and 1 mM H_2_O_2_, compared to control. There was also a non-significant tendency toward a decrease with 0.5 mM H_2_O_2_ compared to 0.05 mM H_2_O_2_-treated cells. p-JNK was not changed by treatment of HUVECs with 0.01–1 mM H_2_O_2_. To determine whether H_2_O_2_-induced suppression of ERK1/2 is a common occurrence in different cell types, immortalized murine aortic endothelial cells (iMAECs) and human brain astroglioma CRT-MG cells were tested. Phosphorylation of ERK1/2 was significantly increased in response to H_2_O_2_ in iMAECs (Fig. 2b), consistent with previous reports showing that H_2_O_2_ induces ERK1/2 phosphorylation. In CRT-MG cells, p-ERK1/2 was also induced by H_2_O_2_ in a concentration-dependent manner (Fig. 2c).

### H_2_O_2_ influences HUVEC apoptosis and monolayer permeability

**Figure 3:**
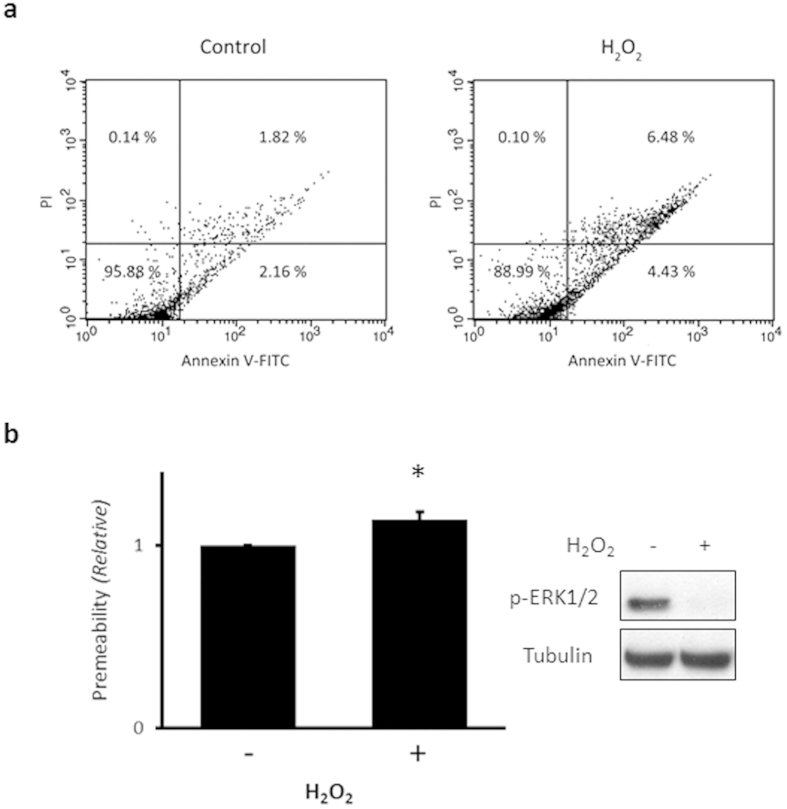
H_2_O_2_ influences HUVEC apoptosis and monolayer permeability. (**a**) HUVECs were treated with 0.5 mM H_2_O_2_ for 15 min, stained with annexin V-FITC and propidium iodide (PI), and then analyzed by fluorescence activated cell sorting (FACS). The proportion of cells in each quadrant was presented. Data are representative of at least three independent experiments. (**b**) HUVECs cultured on collagen-coated inserts were exposed to 0.5 mM H_2_O_2_ for 15 min, and then FITC-dextran solution was added to the inserts. After 20-min incubation at room temperature, the amount of FITC-dextran that passed through the HUVEC monolayer was measured using a fluorescence plate reader. Relative fluorescence intensity is shown. Data are expressed as mean values of three independent samples. *p < 0.05 vs. untreated control. HUVECs cultured on inserts were then lysed in lysis buffer and p-ERK level was analyzed by immunoblotting (right panel). p, phospho.

To test the effect of H_2_O_2_ on HUVEC apoptosis, cells were treated with 0.5 mM H_2_O_2_ for 15 min, stained with annexin V-conjugated FITC and propidium iodine (PI), and then analyzed by fluorescence activated cell sorting (FACS). As shown in Fig. 3a, H_2_O_2_ increased the number of early and late apoptotic cells from 2.16% and 1.82% to 4.43% and 6.48%, respectively. Treating monolayer HUVECs with 0.5 mM H_2_O_2_ for 15 min and measuring permeability using a FITC-dextran solution showed that permeability was significantly increased about 14% above control (Fig. 3b).

### H_2_O_2_ induces Src phosphorylation at tyrosine 530 in HUVECs

**Figure 4:**
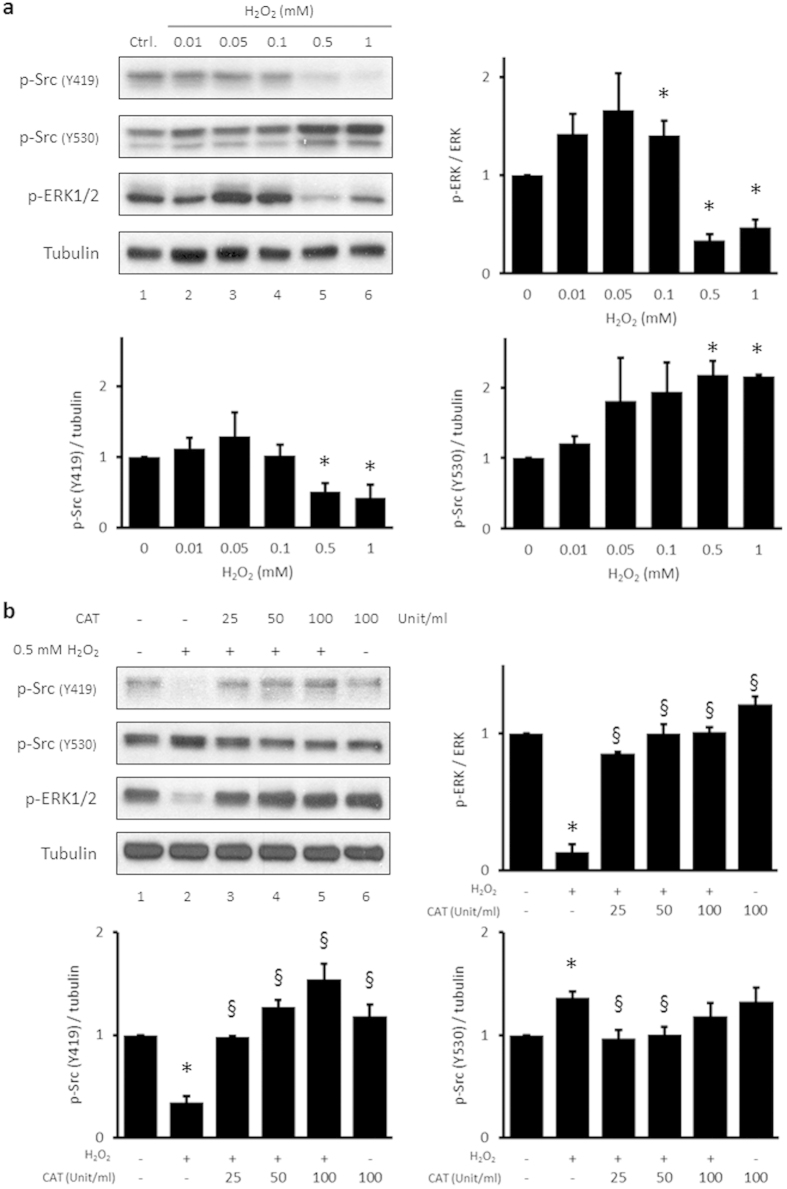
H_2_O_2_ induces Src phosphorylation at tyrosine 530 in HUVECs. (**a**) HUVECs were treated with the indicated concentrations of H_2_O_2_ for 15 min. Whole cell lysates were then extracted and analyzed by immunoblotting. Anti-p-Src Y419, anti-p-Src Y530, anti-p-ERK1/2, anti-ERK1/2, and anti-tubulin antibodies were used. p-ERK1/2 levels were quantified densitometrically and normalized to that of ERK1/2. p-Src Y419 and p-Src Y530 levels were quantified densitometrically and normalized to the level of tubulin. The control level was assigned a value of 1. Data are expressed as mean values ± SEM of three independent samples. *p < 0.05 vs. untreated control. (**b**) Different amounts of catalase were added to HUVECs in the presence or absence of 0.5 mM H_2_O_2_ for 15 min. Whole cell lysates were extracted and analyzed by immunoblotting. p-Src and p-ERK1/2 blots from three independent samples were quantified densitometrically. *p < 0.05 vs. untreated control; ^§^*p* < 0.05 vs. H_2_O_2_ only-treated cells. p, phospho; Y, tyrosine; CAT, catalase.

The phosphorylation of ERK1/2 by H_2_O_2_ is dependent on the activation of Src^29^. To investigate the effect of H_2_O_2_ on Src activation, HUVECs were treated with varied concentrations of H_2_O_2_ for 15 min and immunoblotted with anti-p-Src tyrosine 419 and anti-p-Src tyrosine 530 antibodies. Phosphorylation of Src tyrosine 419 was decreased following treatment with 0.5–1 mM H_2_O_2_ compared with the control (Fig. 4a, first panel, lanes 5–6 vs. 1). In contrast, the phosphorylation of Src tyrosine 530 was significantly increased by these H_2_O_2_ treatments compared to the control (Fig. 4a, second panel, lanes 5–6 vs. 1). To determine whether H_2_O_2_ was directly involved in the phosphorylation of Src tyrosine 530, HUVECs were incubated for 15 min with 25, 50, or 100 units/ml catalase in the presence of 0.5 mM H_2_O_2_. As little as 25 units/ml catalase abrogated H_2_O_2_-induced changes in p-ERK1/2 (Fig. 4b, third panel, lanes 3 and 2), as well as p-Src tyrosine 419 (Fig. 4b, first panel). In contrast to the suppression and recovery patterns of p-ERK1/2 and p-Src tyrosine 419 induced by treatment with H_2_O_2_, p-Src tyrosine 530 was reduced to the control level by co-treatment with 25, 50, and 100 units/ml catalase (Fig. 4b, second panel, lanes 3–6 vs. 2).

### siRNA targeting Csk abrogates the suppression of p-ERK1/2 by H_2_O_2_ by controlling the phosphorylation status of Src tyrosine 530

**Figure 5:**
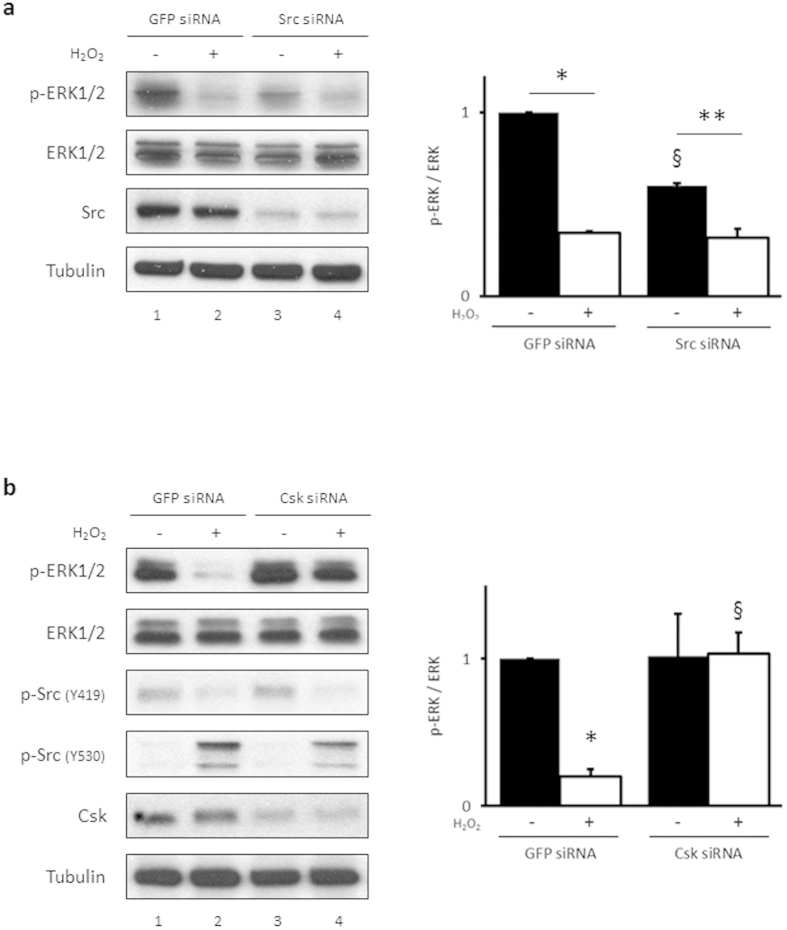
H_2_O_2_-induced suppression of ERK1/2 is dependent on Csk. (**a**) HUVECs were transfected with 100 nM of GFP or Src siRNA using Lipofectamine^TM^ RNAiMAX (1.5 μl/ml). After 48 h, cells were treated with 0.5 mM H_2_O_2_ for 15 min. Whole cell lysates were extracted and analyzed by immunoblotting. Tubulin was used as the loading control. Right panel: p-ERK1/2 levels were quantified densitometrically and normalized to the level of ERK1/2. The control level of p-ERK1/2 expression was assigned a value of 1. Data are expressed as mean values ± SEM of three independent samples. *p < 0.05 between GFP siRNA-transfected control and H_2_O_2_-treated cells; **p < 0.05 between Src siRNA-transfected control and H_2_O_2_-treated cells; ^§^*p* < 0.05 between GFP siRNA-transfected H_2_O_2_-treated cells and Src siRNA-transfected H_2_O_2_-treated cells. (**b**) HUVECs were transfected with 50 nM of GFP or Csk siRNA using Lipofectamine^TM^ RNAiMAX (1.5 μl/ml). After 24 h, cells were treated with 0.5 mM H_2_O_2_ for 15 min. Whole cell lysates were analyzed by immunoblotting. Data are expressed as mean values ± SEM of three independent samples. *p < 0.05 between GFP siRNA-transfected control and H_2_O_2_-treated cells; ^§^*p* < 0.05 between GFP siRNA-transfected H_2_O_2_-treated cells and Csk siRNA-transfected H_2_O_2_-treated cells. p, phospho.

To investigate whether Src was directly involved in the suppression of p-ERK1/2 by H_2_O_2_, HUVECs were transfected with siRNA targeting Src for 48 h, treated with or without H_2_O_2_ for 15 min, and immunoblotted with anti-p-ERK1/2, anti-ERK1/2, and anti-Src antibodies. In the absence of H_2_O_2_, the phosphorylation of ERK1/2 in Src siRNA-transfected cells was significantly decreased compared to GFP siRNA-transfected cells. Interestingly, in the presence of H_2_O_2_, the phosphorylation of ERK1/2 was reduced in both GFP-siRNA transfected and Src-siRNA transfected H_2_O_2_-treated groups, with no significant difference in the phosphorylation level between the two groups (Fig. 5a). From a proportionate perspective, the amount of p-ERK1/2 suppressed by H_2_O_2_ was reduced from 65.4% to 46.9%. Because Csk is the key regulatory molecule that phosphorylates Src tyrosine 530 and inhibits Src kinase activity^34^, HUVECs were transfected with Csk siRNA and the effect of silencing Csk on Src and ERK1/2 was examined in H_2_O_2_-treated HUVECs. After exposure to 0.5 mM H_2_O_2_ for 15 min, the level of p-Src tyrosine 530 was reduced and p-ERK1/2 suppression was significantly abrogated in Csk siRNA-transfected cells, compared to GFP siRNA-transfected cells (Fig. 5b). In contrast to p-Src tyrosine 530, p-Src tyrosine 419 was not affected by Csk siRNA knockdown.

### H_2_O_2_-induced Csk translocation is critical to suppressing p-ERK1/2

**Figure 6:**
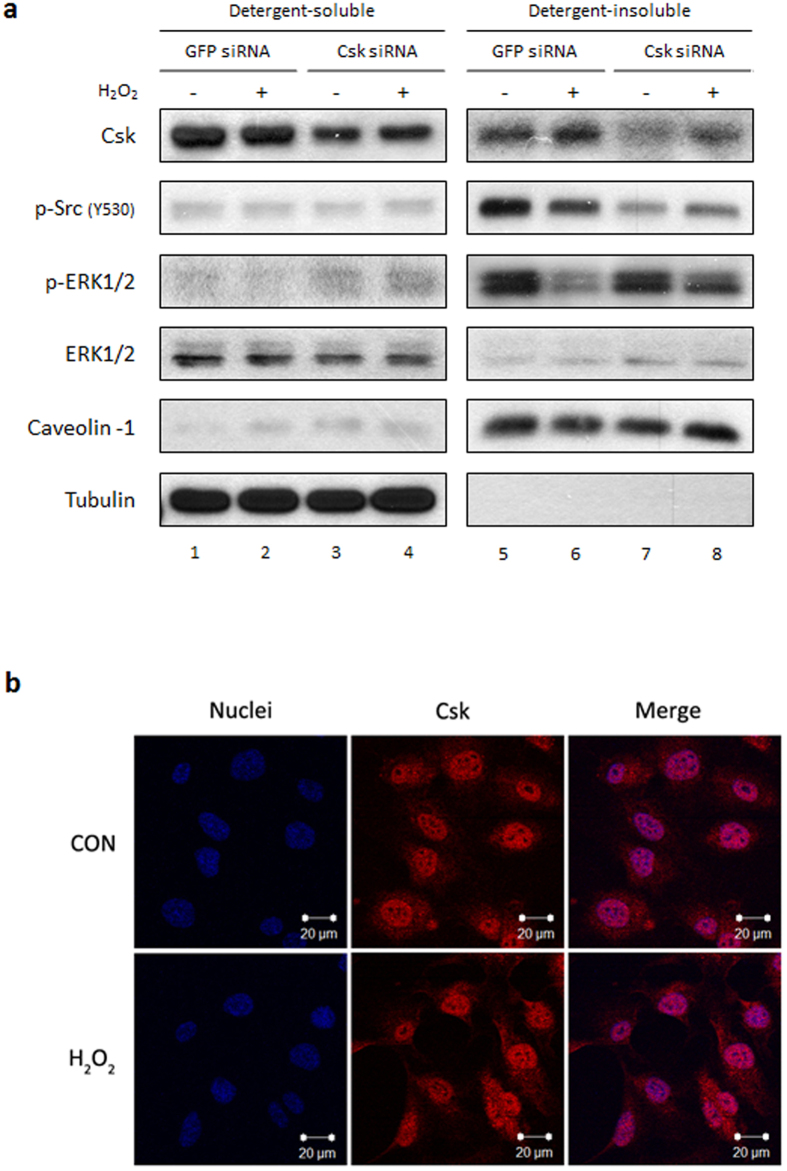
Membrane translocation of Csk is induced by treatment with H_2_O_2_ in HUVECs. (**a**) HUVECs transfected with GFP or Csk siRNA were exposed to 0.5 mM H_2_O_2_, then separated into soluble and insoluble fractions using Brij 58. Each fraction was analyzed by immunoblotting using anti-Csk, anti-p-Src Y530, anti-p-ERK1/2, anti-ERK1/2, anti-caveolin-1, and anti-tubulin antibodies. (**b**) HUVECs were incubated with or without 0.5 mM H_2_O_2_ for 15 min and then fixed with 4% paraformaldehyde. Fixed cells were probed with anti-Csk antibody followed by an Alexa fluor 568-conjugated secondary antibody. Data are representative of at least three independent experiments. (Original magnification × 400). p, phospho; Y, tyrosine; CON, control.

Csk exhibits its inhibitory function on Src through effects on membrane translocation^34^. To confirm the membrane translocation of Csk by treatment with 0.5 mM H_2_O_2_, HUVECs transfected with Csk siRNA were treated with 0.5 mM H_2_O_2_ for 15 min, followed by separation of whole cell lysates into detergent soluble and insoluble fractions using the nonionic detergent Brij 58. Immunoblotting analysis revealed that recruitment of Csk to the insoluble fraction was increased by H_2_O_2_ (Fig. 6a, first panel, lanes 6 and 5). When Csk was suppressed by siRNA knockdown, the amount of Csk and p-Src tyrosine 530 in the insoluble fraction was reduced, while the reduction in p-ERK1/2 in the insoluble fraction caused by H_2_O_2_ was abrogated (Fig. 6a, lanes 5–6 vs. 7–8). Immunofluorescence assay (IFA) data showed that Csk was diffusely distributed throughout the cytoplasm in untreated HUVECs and that a significant quantity of Csk was found at the cell periphery following H_2_O_2_ treatment, although a large quantity of Csk was still present in the cytoplasm (Fig. 6b).

### H_2_O_2_-induced suppression of ERK1/2 phosphorylation is abrogated by physiological laminar flow, but not by oscillatory flow

**Figure 7:**
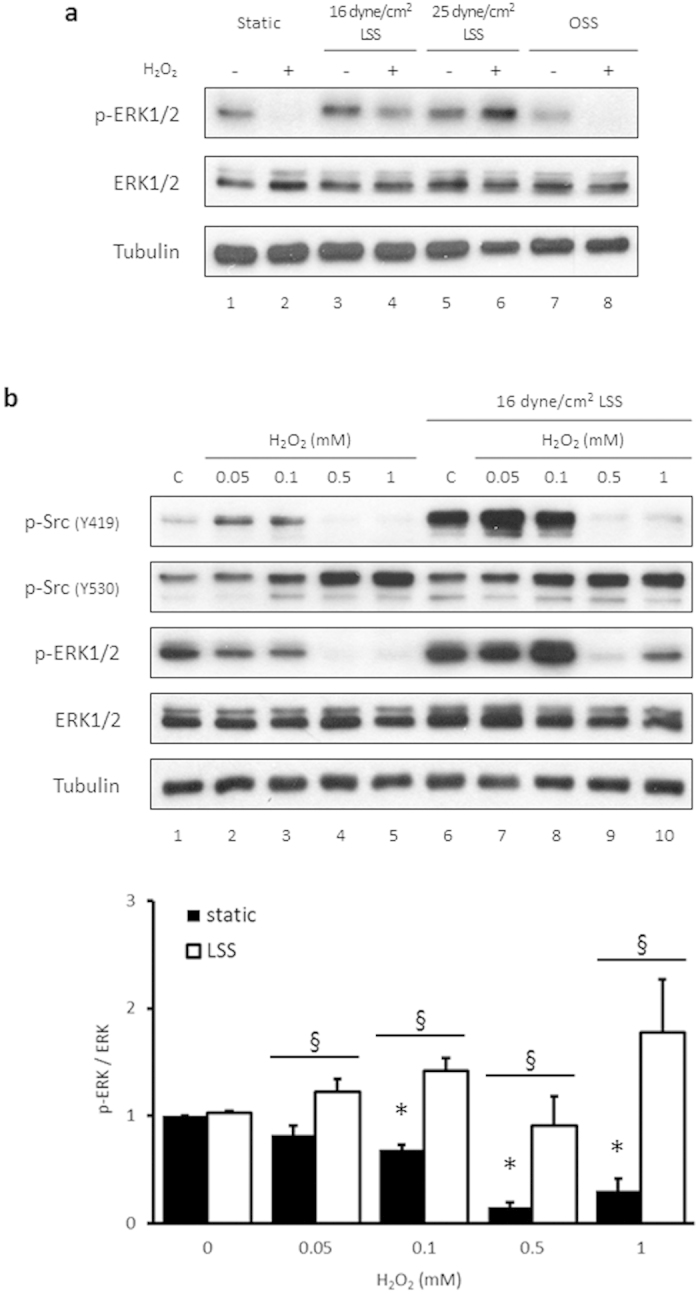
Physiological laminar flow, not oscillatory flow, prevents the H_2_O_2_-induced suppression of ERK1/2 phosphorylation. (**a**) Different strengths of laminar shear or oscillatory shear stress were used on HUVECs for 15 min in the presence or absence of 0.5 mM H_2_O_2_. After treatment, whole cell lysates from each group were extracted and analyzed by immunoblotting. (**b**) HUVECs were exposed to the indicated concentrations of H_2_O_2_ for 15 min with or without 16 dyne/cm^2^ laminar shear stress. Whole cell lysates were extracted and analyzed by immunoblotting. Bottom panel: p-ERK1/2 levels were quantified densitometrically and normalized to the level of ERK1/2. The control level of p-ERK1/2 expression was assigned a value of 1. Data are expressed as mean values ± SEM of three independent samples. *p < 0.05 vs. H_2_O_2_-untreated static control; ^§^*p* < 0.05 between static cells and LSS-treated cells at each concentration of H_2_O_2_. LSS, laminar shear stress; OSS, oscillatory shear stress; p, phospho.

In a normal physiological state, endothelial cells lining the inner surface of blood vessels are exposed to mechanical shear stress from the circulating blood flow^14^. To determine whether H_2_O_2_-induced suppression of p-ERK1/2 occurs in the presence of shear stress, HUVECs were treated with or without 0.5 mM H_2_O_2_ for 15 min under conditions of laminar shear stress (16 and 25 dyne/cm^2^) or oscillatory shear stress. Interestingly, H_2_O_2_-induced suppression of p-ERK1/2 was abolished in the presence of laminar shear stress (Fig. 7a, first panel, lanes 4 and 6 vs. 2). In contrast, suppression of ERK1/2 phosphorylation induced by H_2_O_2_ was unaffected by oscillatory shear stress. HUVECs were also treated with various concentrations of H_2_O_2_ for 15 min in the presence or absence of 16 dyne/cm^2^ laminar shear stress. Under these conditions, ERK1/2 phosphorylation was increased and the suppression of p-ERK1/2 by H_2_O_2_ was significantly diminished, especially with 1 mM H_2_O_2_ (Fig. 7b, third panel, lanes 10 and 5). Interestingly, phosphorylation of Src tyrosine 530 was not changed by laminar shear stress but only by H_2_O_2_, while the phosphorylation of Src tyrosine 419 was increased by laminar shear stress and significantly reduced by H_2_O_2_.

## Discussion

The results of the current study showed that phosphorylation of ERK1/2 in HUVECs are suppressed by H_2_O_2_ at particular concentrations and times, and that this suppression is dependent on Csk translocation and subsequent phosphorylation of Src tyrosine 530. Physiological laminar flow, but not oscillatory shear stress, abrogates the suppression of p-ERK1/2 by H_2_O_2_. The suppression of p-ERK1/2 by H_2_O_2_ seems to conflict with other reports showing that H_2_O_2_ induces the phosphorylation of ERK1/2^7^,^25^,^36^,^37^,^38^,^39^. However, our findings differ from previous reports in that the suppression of p-ERK1/2 is specific to HUVECs. When either murine endothelial or astroglioma cells were treated with 0.5 mM H_2_O_2_ for 15 min, p-ERK1/2 was increased (Fig. 2b,c). This can explain the finding of Yang *et al.* that ERK1/2 phosphorylation is increased in bovine aortic endothelial cells treated in a similar manner^37^. The reason for the different response in HUVECs may be related to the high basal phosphorylation of ERK1/2 in these cells compared to other cell types. As a result, H_2_O_2_-induced suppression of p-ERK1/2 may be easier to detect in HUVECs. In addition to cell type, time and concentration are important factors in the effects of H_2_O_2_. Suppression of ERK1/2 phosphorylation by 0.5 mM H_2_O_2_ occurs in a relatively short time range (within 15 min) and rebounds at 30 min when there is a high level of p-ERK1/2 compared to untreated controls (Fig. 1b). The increased p-ERK1/2 observed at 30 min is consistent with the report of Kevil *et al.* showing that ERK1/2 is activated by 1 mM H_2_O_2_ after 30 min in HUVECs^7^. In addition, this fact suggests that transient ERK1/2 suppression provides a window during which cells can prepare for the later activation of ERK1/2. Suppression of p-ERK1/2 occurred only at H_2_O_2_ concentrations between 0.25 and 1 mM, while ERK1/2 phosphorylation was slightly increased at 0.1 mM H_2_O_2_ (Fig. 1a). This is consistent with other reports showing ERK1/2 activation at relatively low concentrations of H_2_O_2_^36^,^38^. This result suggests that both concentration and time are important factors of suppression of p-ERK1/2 by H_2_O_2_.

ERK1/2 is a well-studied signaling molecule in various cell types, including endothelial cells. The phosphorylation of ERK1/2 activates many downstream signaling molecules, and induces cell proliferation and protection against oxidative stress^25^. However, the role of ERK1/2, and the effect of ERK1/2 phosphorylation, has been controversial in endothelial cell biology. ERK1/2 mediates the loss of cell-to-cell junctional integrity and the increased cell permeability induced by H_2_O_2_^7^. When ERK1/2 activity is suppressed by the pharmacological inhibitor PD98059, H_2_O_2_-evoked increases in permeability are prevented. ERK1/2 activation also activates pro-survival signaling and prevents the apoptosis induced by H_2_O_2_. Thus, apoptosis is increased when ERK1/2 function is blocked^17^,^26^,^37^. On the other hand, activation of ERK1/2 contributes to cell death induced by cerebral ischemia. Phosphorylation level of ERK1/2 increases by oxygen-glucose deprivation (OGD), *in vitro* ischemic model, and a specific inhibitor of MEK protects cell injury against OGD^40^,^41^. In our results, treatment with 0.5 mM H_2_O_2_ increased apoptotic cell death and increased monolayer permeability in HUVECs. This result is consistent with previous reports that H_2_O_2_ increases endothelial cell permeability but conflicts with the finding that the activation of ERK1/2 contributes to the disruption of endothelial cell integrity^7^,^42^. This discrepancy might due to different time and concentration of H_2_O_2_.

Because MAPK subfamilies interact with each other, the levels of p-p38 and p-JNK were measured. Although ERK1/2 phosphorylation was decreased by 0.5 mM H_2_O_2_, p38 and JNK showed no significant change in phosphorylation (Fig. 2a). Interestingly, although the p-p38 level after treatment with 0.5 mM H_2_O_2_ was slightly decreased compared to the group treated with 0.05 mM H_2_O_2_, the much larger suppression seen with p-ERK1/2 was not observed. Generally, the function of ERK1/2 in eukaryotic cells differs from the function of p38 and JNK. While ERK1/2 primarily participates in cell growth and proliferation, p38 and JNK have a role in stress and inflammatory responses rather than mitogen responses^43^. Wang *et al.* reported that ERK1/2 had an anti-apoptotic effect while p38 did not affect H_2_O_2_-mediated apoptosis and JNK participated in pro-apoptotic signaling^17^.

Significant suppression of p-ERK1/2 by H_2_O_2_ was related with the inactivation of Src through the phosphorylation of Src tyrosine 530. When phosphorylation of ERK1/2 was suppressed by 0.5 mM H_2_O_2_, phosphorylation of Src tyrosine 530 was increased while phosphorylation of Src tyrosine 419 was reduced (Fig. 4a,b). Src activates various molecules involved in endothelial cell permeability and induces vascular leakage^31^. Src is regulated by two major phosphorylation sites, tyrosine 419 and tyrosine 530. When Src tyrosine 419 is phosphorylated, Src undergoes a conformational change resulting in activation; however, when Src tyrosine 530 is phosphorylated, Src is inactive and maintains a “closed” conformation^32^. Dephosphorylating Src at tyrosine 530 is as important to Src activation as is Src tyrosine 419 phosphorylation. In human colon cancer cells, increased Src activation is mediated by the reduction of Src tyrosine 530 phosphorylation rather than Src tyrosine 419 phosphorylation^44^,^45^. When Src expression was silenced by siRNA, the percentage of reduction in H_2_O_2_-mediated suppression of ERK1/2 phosphorylation was decreased from 65.4% to 46.9% (Fig. 5a). Although the difference in the percentage of reduction between the GFP siRNA- and Src siRNA-transfected H_2_O_2_-treated groups was relatively small, Src knockdown reduced the suppression of p-ERK1/2 by H_2_O_2_, perhaps because of changes in the Src expression level. The quantity of Src is reduced in Src knockdown cells, resulting in a decrease in the quantity of p-Src tyrosine 530 induced by H_2_O_2_. Thus, both Src activity and the suppression of ERK1/2 in the presence of H_2_O_2_ were decreased by Src knockdown. The observed difference in the efficacy of Src and Csk knockdown in reversing H_2_O_2_-mediated suppression of ERK1/2 phosphorylation might be due to the complicated mechanism of action of Src. The knockdown of Src induces depletion of two major phosphorylation sites, tyrosine 419 and tyrosine 530, which may result in a loss of counteraction.

Csk is a negative regulator of Src that phosphorylates Src tyrosine 530 and maintains Src in the inactive state. Membrane translocation and co-localization with Src are required for Csk functioning^33^,^34^. Suppression of ERK1/2 phosphorylation was abrogated by Csk siRNA knockdown (Fig. 5b). This indicates the dependency of Csk and Src tyrosine 530 for the suppression of p-ERK1/2 by H_2_O_2_. IFA and immunoblotting data showed that translocation of Csk to the cell membrane occurred in response to H_2_O_2_ (Fig. 6a,b). In addition, when the movement of Csk to the detergent-insoluble fraction was blocked by silencing Csk, the phosphorylation of Src tyrosine 530 was decreased and suppression of p-ERK1/2 by H_2_O_2_ was abolished (Fig. 6a). This finding suggests that the H_2_O_2_-induced suppression of pERK1/2 is dependent on the membrane translocation of Csk and subsequent phosphorylation of Src tyrosine 530. Interestingly, Cao *et al.* reported that both Src and Csk are activated by oxidative stress and this paradoxical activation of Csk with Src may be important to stabilize Src in its basal state^46^. Activation of Csk and subsequent inactivation of Src in this study may largely affect the H_2_O_2_-mediated ERK1/2 signaling pathway.

Endothelial cells lining the vascular wall are continuously exposed to mechanical force because of blood pressure and flow. Laminar shear stress, the tangential force created by blood flow, plays an important role in endothelial function and helps prevent atherosclerosis^13^,^47^. ERK1/2 has an important role in the various events mediated by shear stress, which activates both Src and ERK1/2^13^. When different strengths of laminar shear stress were applied together with 0.5 mM H_2_O_2_ for 15 min, suppression of ERK1/2 was significantly abrogated (Fig. 7a). However, there was no change in H_2_O_2_-induced ERK1/2 suppression in either the static or oscillatory shear stress groups treated with H_2_O_2_. Interestingly, laminar shear stress increased the phosphorylation levels of Src tyrosine 419 as well as ERK1/2 (Fig. 7b). It has been reported that Src tyrosine 419 phosphorylation, Src tyrosine 530 dephosphorylation, and Src activation occur with laminar shear stress as opposed to oscillatory shear stress^48^. In addition, Tian *et al.* reported that H_2_O_2_ primes the shear stress-induced activation of eNOS^49^. Thus, nitric oxide production is increased in response to treatment of cells with shear stress and H_2_O_2_ compared to H_2_O_2_ alone-treated cells. These results suggest that, in a normal endothelial cell environment where shear stress exists continuously, physiological laminar flow could abolish the H_2_O_2_-induced suppression of p-ERK1/2.

**Figure 8:**
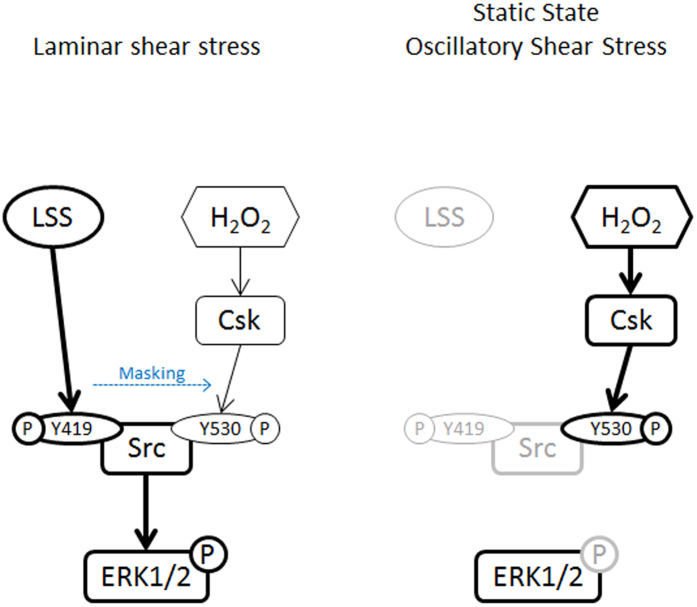
Potential mechanism of ERK1/2 suppression by H_2_O_2_ in HUVECs. During normal physiological shear stress, laminar flow induces phosphorylation of Src tyrosine 419, which masks Csk- and Src tyrosine 530-mediated suppression of p-ERK1/2 in the presence of H_2_O_2_. As a result, phosphorylation of ERK1/2 is not suppressed by H_2_O_2_. However, when physiological laminar flow is disrupted, such as during a static status or oscillatory flow, H_2_O_2_ induces Csk translocation and Src tyrosine 530 phosphorylation. This leads to the inactivation of Src and subsequent suppression of ERK1/2 phosphorylation in HUVECs.

In conclusion, this is the first report describing that, in normal environments with physiological shear stress, laminar flow masks Csk and Src tyrosine 530-mediated suppression of ERK1/2 phosphorylation, which is induced by H_2_O_2_. As a result, p-ERK1/2 is not significantly altered by H_2_O_2_ in the presence of laminar shear stress. However, when physiological laminar flow is disrupted because of regional features such as a bifurcation point, atherosclerosis, or an obstruction, the H_2_O_2_-induced suppression of ERK1/2 could occur through Csk translocation and subsequent Src tyrosine 530 phosphorylation (Fig. 8). Further studies are needed to determine how HUVECs recognize concentration differences of H_2_O_2_ and activate different signaling pathways. This study, however, contributes to broaden our understanding of signaling mechanisms in endothelial cells against oxidative stress and shear stress.

## Methods

### Cell culture

Human umbilical vein endothelial cells (HUVECs) were kindly provided by Dr. Kihwan Kwon (Ewha Womans University, Seoul, Korea). HUVECs were maintained in Medium 200 (Life Technologies, Carlsbad, CA, USA) with 5% fetal bovine serum (FBS), 1% penicillin-streptomycin, and low-serum growth supplement (LSGS; Life Technologies) and all dishes and flasks for HUVEC are pre-coated by 2% gelatin (Wako Pure Chemical Industries, Ltd., Osaka, Japan) solution. HUVECs in passage 5 to 8 were used in all experiments. Immortalized murine aortic endothelial cells (iMAECs) were grown in Dulbecco’s modified essential media (DMEM; GE Healthcare Life Science, Buckinghamshire, UK.) containing 100 μg/ml endothelial cell growth supplement (Sigma, St. Louis, MO, USA), 1x non-essential Eagle’s amino acid, 10% fetal bovine serum and 1% penicillin-streptomycin. Human astroglioma cell line CRT-MG were grown in DMEM (WelGENE, Daegu, Korea) with 10% fetal bovine serum and 1% penicillin-streptomycin.

### Reagents and antibodies

H_2_O_2_ and catalase were purchased from Sigma. Antibodies against p-ERK1/2, ERK1/2, p-Src Y419, p-Src Y530 and Src were obtained from Cell Signaling (Beverly, MA, USA). Anti-Csk, anti-p-p38, anti-p38, anti-p-JNK, anti-JNK antibodies were obtained from Santa Cruz Technology (Santa Cruz, CA, USA). Antibody against caveolin-1 was obtained from BD Biosciences (San Diego, CA, USA). Tubulin antibody was obtained from Sigma. All Horseradish peroxidase (HRP)-conjugated secondary antibodies for immunoblotting were obtained from Santa Cruz Technology. Alexa flour 568-conjugated rabbit secondary antibody was obtained from Life Technologies.

### Shear stress exposure

Cone and plate viscometer was used to apply shear stress to HUVEC *in vitro*. Confluent cells in 60-mm dishes were exposed to fluid shear stress. Unidirectional steady flow (shear stress of 16 dyne/cm^2^ or 25 dyne/cm^2^) for laminar shear stress or a bidirectional disturbed flow (shear stress of ± 5 dyne/cm^2^) for oscillatory shear stress was exposed to confluent HUVECs.

### Small interfering RNA (siRNA) transfection

Cells were transfected with green fluorescence protein (GFP) siRNA from Samchully Pharm Co. Ltd. (Seoul, Korea), 50 nM of human Csk siRNA from Bioneer (Daejeon, Korea) or 100 nM of SMART pools of human Src siRNA from GE Healthcare Life Science using Lipofectamine^TM^ RNAiMAX reagent (Life Technologies) according to the manufacturer’s protocol. After transfection, HUVECs were incubated in media containing 5% FBS, 1% penicillin-streptomycin, and LSGS for 24 h (in the case of Csk) or 48 h (in the case of Src) and then exposed to 0.5 mM H_2_O_2_ for 15 min.

### Western blot analysis

Cells were washed once with phosphate-buffered saline (PBS) and lysed by ice-cold radioimmunoprecipitation assay (RIPA) buffer containing *Xpert* Protease Inhibitor Cocktail (GenDEPOT, Inc., Barker, TX, USA) and 0.5 mM sodium orthovanadate (Na_3_VO_4_). Lysates were centrifuged (12,000 g) at 4 °C for 30 min and then supernatants were used for immunoblot analysis. Proteins were separated by 10% or 12% SDS-PAGE and transferred to polyvinylidene difluoride (PVDF) membrane. The membranes were blocked by 5% skim milk in Tris-buffered saline containing 0.1% Tween-20 (TBS-T) and probed with primary antibodies followed by HRP-conjugated specific secondary antibodies. Immunoreactive blots were developed using ECL chemiluminescence system (Amersham, Buckinghamshire, UK) and exposed to blue X-ray film (AGFA, Mortsel, Belgium).

### Vascular permeability assay

Endothelial cell permeability was measured using 24-well *In vitro* Vascular Permeability Assay (Millipore, Darmstadt, Germany) according to manufacturer’s protocol. 2 × 10^5^ of HUVECs were seeded onto collagen-coated inserts, and incubated for 72 h monolayer formation time. After incubation, 0.5 mM H_2_O_2_ were treated to HUVEC monolayer for 15 min, and then FITC-dextran solution was added to each inserts. Inserts and receivers protected from light were further incubated for 20 min. At the end of the incubation time, 100 μl of media in each receiver tray was harvested and the amount of FITC-dextran which crossed the monolayer was measured using Synergy H1 Hybrid Multi-Mode Microplate Reader (BioTek Instruments, Inc., Winooski, VT, USA). After measurement, cell monolayer integrity was checked using Cell Stain (Millipore).

### Cell apoptosis assay

The HUVEC apoptosis was examined using FITC Annexin V Apoptosis Detection Kit II (BD Biosciences) according to the manufacturer’s protocol. Confluent HUVECs were treated with 0.5 mM H_2_O_2_ for 15 min, and then harvested cells were stained with annexin Ⅴ-FITC and propidium iodine (PI) in the dark at room temperature for 15 min. Cells were analyzed by FACSCaliburTM flow cytometry (BD Biosciences).

### Isolation of detergent-insoluble fraction

A detergent-insoluble fraction was isolated according to a protocol previously described^50^. Cells were washed once with PBS and lysed with HEPES buffer (10 mM sodium HEPES, pH 7.5, 150 mM NaCl, 5 mM EDTA, protease inhibitor mixture and 0.5 mM Na_3_VO_4_) containing 0.5% Brij 58. Cell lysates were centrifuged at 12,000 g for 30 min at 4 °C, and then supernatants were obtained and used as soluble fractions in following experiments. The pellets were washed once with cold HEPES buffer without detergent, solubilized with lysis buffer (50 mM Tris–HCl, pH 7.4, 0.1% SDS, 0.5% sodium deoxycholate, 1% Nonidet P-40, 1 mM EDTA, 1 mM EGTA, protease inhibitor mixture and 0.5 mM Na_3_VO_4_), and centrifuged at 12,000 g for 30 min at 4 °C. These supernatants were used as insoluble fractions. Each fraction was analyzed by immunoblotting.

### Immunofluorescence assay

HUVECs cultured on poly-D-lysine-coated coverslips were washed once with ice-cold PBS and fixed with 4% paraformaldehyde for 1 h at room temperature. Fixed cells were incubated in PBS-T (PBS with 0.25% Triton X-100) for 15 min, and permeable cells were blocked by 1% BSA in PBS-T for 1 h. Cells were probed with anti-Csk antibody overnight, washed with ice-cold PBS twice and then probed by Alexa flour 568-conjugated secondary antibody for 90 min. Cells were mounted in mounting solution (Life Technologies) containing DAPI and observed using confocal microscope (Carl Zeiss, Oberkochen, Germeny).

### Statistical analysis

Mean ± standard error of the mean (SEM) were showed in all bar graphs from at least three independent experiments, and statistical significance was analyzed by student t-test using the SPSS software version 20.0 (SPSS Inc., Chicago, IL, USA). A value of *p* < 0.05 was regarded statistically significant.

## Additional Information

**How to cite this article**: Jeon, B. K. *et al.* Csk-Induced Phosphorylation of Src at Tyrosine 530 is Essential for H_2_O_2_-Mediated Suppression of ERK1/2 in Human Umbilical Vein Endothelial Cells. *Sci. Rep.* **5**, 12725; doi: 10.1038/srep12725 (2015).

## Supplementary Material

Supplementary Information
