# Supplementary material for: Csk-Induced Phosphorylation of Src at Tyrosine 530 is Essential for H2O2-Mediated Suppression of ERK1/2 in Human Umbilical Vein Endothelial Cells
Source: Sci Rep. 2015 Aug 3;5:12725. doi: 10.1038/srep12725 (PMC4522603; doi:10.1038/srep12725)
Supplement: Supplementary Information [file srep12725-s1.doc]

**Supplementary Information**

**Csk-Induced Phosphorylation of Src at Tyrosine 530 is Essential for H_2_O_2_-Mediated Suppression of ERK1/2 in Human Umbilical Vein Endothelial Cells**

Bo Kyung Jeon^a,b^, Kihwan Kwon^c^, Jihee Lee Kang^a,b^, Youn-Hee Choi^a,b^

^a^Department of Physiology, School of Medicine, Ewha Womans University, Seoul, Korea; ^b^Tissue Injury Defense Research Center, School of Medicine, Ewha Womans University, Seoul, Korea; ^c^Department of Internal Medicine, Division of Cardiology, School of Medicine, Ewha Womans University, Seoul, Korea


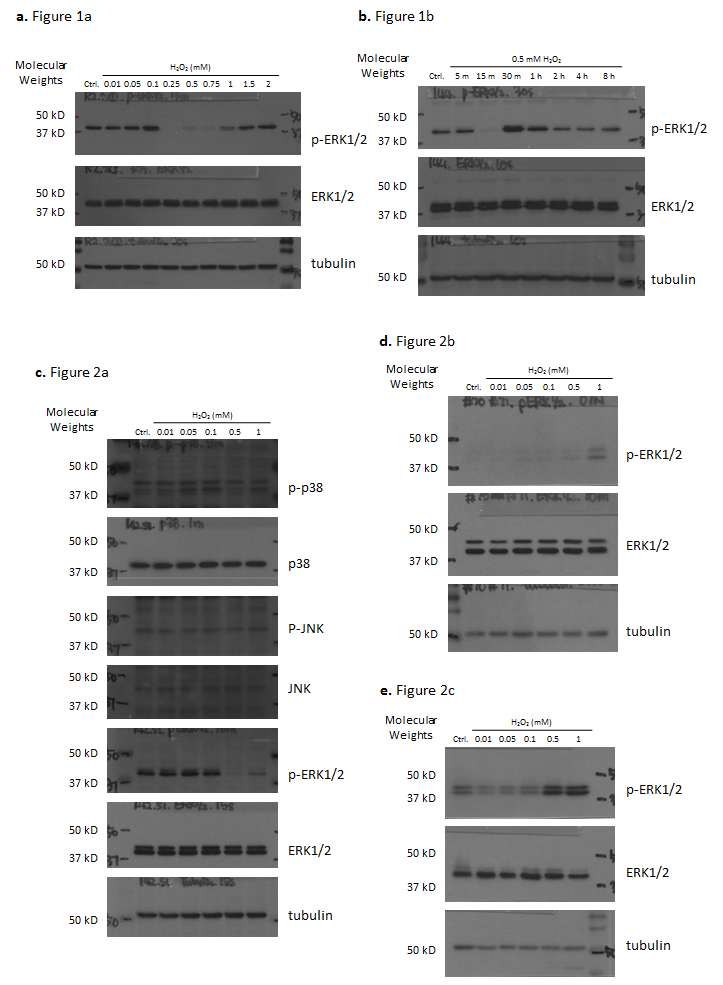


Supplementary Figure 1: Full-length images of the blots presented in the Figure 1 and 2.

A: Full-length images of Figure 1a show that phosphorylation of ERK1/2 was reduced by 0.25-1 mM H_2_O_2_. HUVEC were treated with 0-2 mM H_2_O_2_ for 15 min and whole cell lysates were analyzed by immunoblotting. Tubulin was used as the loading control. p, phospho

B: Full-length images of Figure 1b show that p-ERK1/2 was significantly reduced by H_2_O_2_ at 15 min. HUVEC were treated with 0.5 mM H_2_O_2_ for the indicated times. Whole cell lysates were extracted and analyzed by immunoblotting. Tubulin was used as the loading control. p, phospho

C: Full-length images of Figure 2a show that the phosphorylation of p38 and JNK were not affected by H_2_O_2_. HUVEC were treated with 0-1 mM H_2_O_2_ for 15 min and whole cell lysates were analyzed by immunoblotting against p-p38, p38, p-JNK, JNK, p-ERK1/2 and ERK1/2. Tubulin was used as the loading control. p, phospho

D, E: Full-length images of Figure 2b and 2c show that phosphorylation of ERK1/2 was increased by H_2_O_2_ in iMAEC (D, 2b) and CRT-MG (E, 2c). iMAEC and CRT-MG were treated with 0-1 mM H_2_O_2_ for 15 min and whole cell lysates were analyzed by immunoblotting. Tubulin was used as the loading control. p, phospho


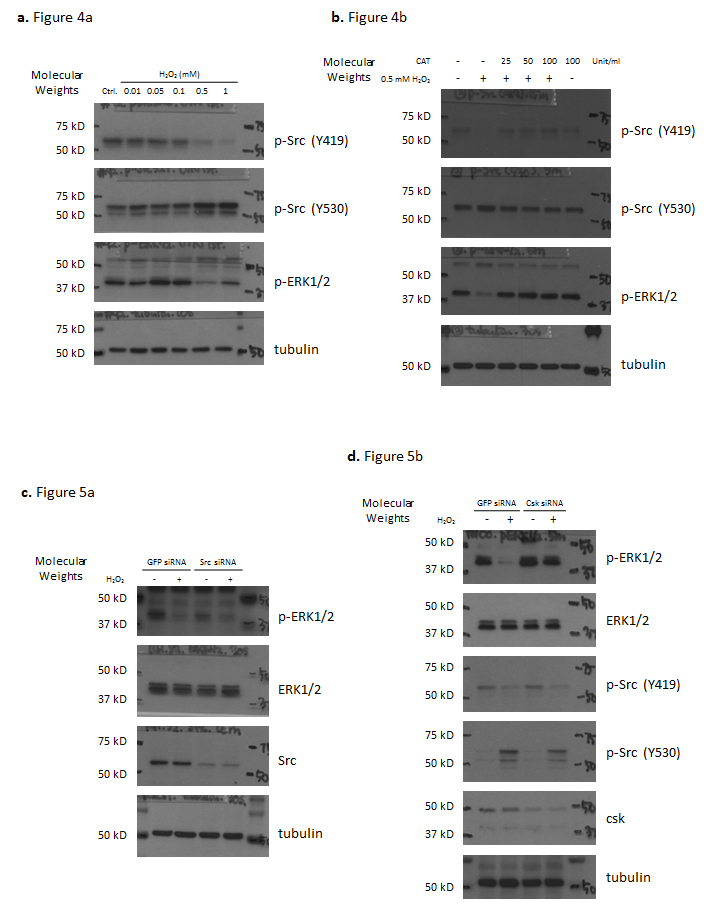


Supplementary Figure 2: Full-length images of the blots presented in the Figure 4 and 5.

A: Full-length images of Figure 4a show that phosphorylation of Src at tyrosine 530 by H_2_O_2_ inversely correlated with phosphorylation of ERK1/2. HUVEC were treated with indicated concentrations of H_2_O_2_ for 15 min. Whole cell lysates were analyzed by immunoblotting. Anti-p-Src Y419, anti-p-Src Y530, anti-p-ERK1/2, and anti-ERK1/2 antibodies were used. Tubulin was used as the loading control. p, phospho; Y, tyrosine

B: Full-length images of Figure 4b show that catalase abrogated H_2_O_2_-induced changes in p-ERK1/2. HUVEC were treated with several amounts of catalase for 15 min in the presence of 0.5 mM H_2_O_2_. Whole cell lysates were analyzed by immunoblotting. Tubulin was used as the loading control. p, phospho; Y, tyrosine; CAT, catalase

C: Full-length images of Figure 5a show that Src knockdown decreased the proportion of p-ERK1/2 suppressed by H_2_O_2_. HUVEC were transfected with 100 nM of GFP or Src siRNA. After 48 h, cells were treated with 0.5 mM H_2_O_2_ for 15 min. Whole cell lysates were extracted and analyzed by immunoblotting. Tubulin was used as the loading control. p, phospho

D: Full-length images of Figure 5b show that Csk knockdown abrogates the suppression of p-ERK1/2 by H_2_O_2_. HUVEC were transfected with 50 nM of GFP or Csk siRNA. After 24 h, cells were treated with 0.5 mM H_2_O_2_ for 15 min. Whole cell lysates were extracted and analyzed by immunoblotting. Tubulin was used as the loading control. p, phospho; Y, tyrosine


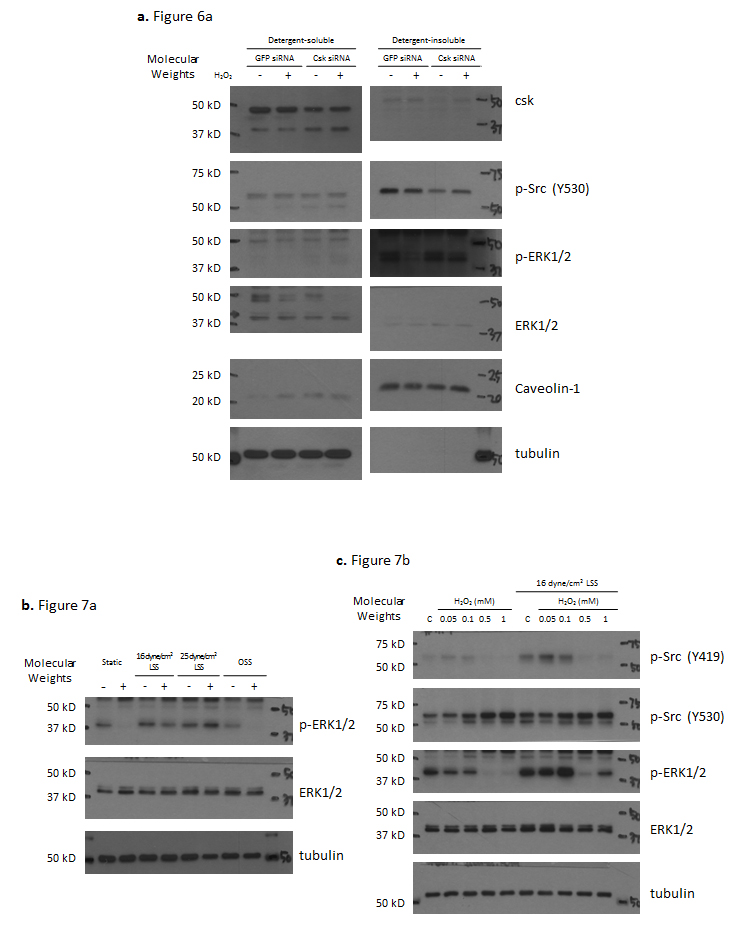


Supplementary Figure 3: Full-length images of the blots presented in the Figure 6 and 7.

A: Full-length images of Figure 6a show that Csk translocation induced by H_2_O_2_ and subsequent changes of p-Src and p-ERK1/2. HUVECs transfected with GFP or Csk siRNA were exposed to 0.5 mM H_2_O_2_. Cell lysates were separated into soluble and insoluble fraction using Brij58 and each fraction was analyzed by immunoblotting. Tubulin and caveolin-1 were used as the loading control. p, phospho; Y, tyrosine

B: Full-length images of Figure 7a show that H_2_O_2_-induced suppression of p-ERK1/2 was abrogated by physiological laminar flow. HUVEC were treated with 0.5 mM H_2_O_2_ under different strengths of laminar shear or oscillatory shear stress. Whole cell lysates were analyzed by immunoblotting. Tubulin was used as the loading control. p, phospho; LSS, laminar shear stress; OSS, oscillatory shear stress

C: Full-length images of Figure 7b show the relation between the level of p-ERK1/2 and p-Src changed by H_2_O_2_ and shear stress co-treatment. HUVEC were treated with indicate concentrations of H_2_O_2_ for 15 min in the presence or absence of laminar shear stress. Whole cell lysates were analyzed by immunoblotting. Tubulin was used as the loading control. p, phospho; Y, tyrosine; LSS, laminar shear stress; OSS, oscillatory shear stress
